# Putative anoikis resistant subpopulations are enriched in lymph node metastases and indicate adverse prognosis in colorectal carcinoma

**DOI:** 10.1007/s10585-022-10184-5

**Published:** 2022-08-26

**Authors:** Taneli T. Mattila, Madhura Patankar, Juha P. Väyrynen, Kai Klintrup, Jyrki Mäkelä, Anne Tuomisto, Pentti Nieminen, Markus J. Mäkinen, Tuomo J. Karttunen

**Affiliations:** 1 Department of Pathology, Cancer and Translational Medicine Research Unit, University of Oulu, POB 5000, 90014 Oulu, Finland; 2 Department of Pathology, Oulu University Hospital and Medical Research Center Oulu, POB 21, 90029 Oulu, Finland; 3 Division of Gastroenterology and Hepatology, Department of Medicine, Keck School of Medicine of USC, Los Angeles, CA 90089-0110 USA; 4 Department of Surgery, Oulu University Hospital and Medical Research Center Oulu, POB 21, 90029 Oulu, Finland; 5 Department of Surgery, Research Unit of Surgery, Anesthesia and Intensive Care, University of Oulu, POB 5000, 90014 Oulu, Finland; 6 Medical Informatics and Data Analysis Research Group, University of Oulu, P.O. Box 5000, 90014 Oulu, Finland

**Keywords:** Anoikis, Colorectal carcinoma, Cribriform, Solid, Micropapillary

## Abstract

Anoikis refers to apoptosis induced by the loss of contact with the extracellular matrix. Anoikis resistance is essential for metastasis. We have recently shown that it is possible to quantitatively evaluate putative anoikis resistant (AR) subpopulations in colorectal carcinoma (CRC). Abundance of these multi-cell structures is an independent marker of adverse prognosis. Here, we have quantified putative AR subpopulations in lymph node (LN) metastases of CRC and evaluated their prognostic value and relationship with the characteristics of primary tumors. A case series included 137 unselected CRC patients, 54 with LN metastases. Areal densities (structures/mm^2^) of putative AR structures in primary tumors had been analyzed previously and now were determined from all nodal metastases (n = 183). Areal density of putative AR structures was higher in LN metastases than in primary tumors. Variation of the areal density within different LN metastases of a single patient was lower than between metastases of different patients. Abundance of putative AR structures in LN metastases was associated with shorter cancer specific survival (p = 0.013), and this association was independent of T and N stages. Abundance of putative AR structures in primary tumors and LN metastases had a cumulative adverse effect on prognosis. Enrichment of putative AR subpopulations in LN metastases suggest that in metastasis formation, there is a selection favoring cells capable of forming these structures. Higher intra-case constancy relative to inter-case variation suggests that such selection is stable in metastasis development. Our findings indirectly support the biological validity of our concept of putative AR structures.

## Introduction

Colorectal carcinoma (CRC) causes 1 in 10 of global cancer deaths [1]. About 15–30 percent of patients with colorectal cancer have synchronous or metachronous metastases [2]. The 5-year survival for patients with locally advanced (stage III) disease is poorer than that of patients without lymph node (LN) metastasis (stage II) (59.5% versus 82.5%) [3]. To improve prognosis of locally advanced CRC, advances in treatment, as well as prognostic and predictive factors are needed [4]. However, there has been only minor focus on the prognostic features present in metastases [5].

Anoikis is a subtype of programmed cell death, where an epithelial cell dies after detachment from the extracellular matrix (ECM) [6]. Physiologically, anoikis prevents the colonization of detached cells elsewhere in the body, and during metastasis formation, resisting anoikis is advantageous. Anoikis resistance is needed as tumor cells detach from their site of origin and disseminate intravascularly, and also when they finally interact with the foreign ECM [6]. Hence, the ability to resist anoikis is a fundamental mechanism involved in metastasis.

There exists some indirect evidence for the presence of anoikis resistance in human carcinomas while direct evidence is limited. The evaluation of anoikis resistance has previously been limited to in vitro cell culture experiments [7], and anoikis resistance related biomarkers have been proposed [8–11]**.** However, it has not been possible to evaluate anoikis resistance by using conventional tissue sections. We have recently shown that transfection of Caco-2 cells with mutant *KRAS* or *BRAF* genes modifies both the measured anoikis resistance and induce a characteristic change in the 3D cell culture growth pattern. We found that native Caco-2 cells show a low level of anoikis resistance and in 3D cultures form cysts with a single layer of columnar cells, all with a contact with the ECM. In contrast, the transfected Caco-2 cells show a high level of anoikis resistance, and in 3D cultures form partially filled cysts with the inner cells detached from the ECM but still maintaining resistance to anoikis [12]. In search of histopathological features consistent with anoikis resistance in actual carcinomas, we have shown that most cells in micropapillary structures (MIP), cribriform structures and solid structures are devoid of contact with ECM proteins and yet do not show increased apoptosis rate [13], and thus represent putative anoikis resistant (AR) subpopulations. We have described a practical method to quantify these putative AR structures in conventional histological tumor tissue sections and shown that a high areal density of such structures in primary tumors indicates adverse prognosis in CRC [13]**.**

Both the significance of putative AR structures as a marker of true anoikis resistance and the prognostic significance of these structures in CRC need to be confirmed. Interestingly, there are previously described growth patterns similar to the putative AR structures that are associated with worse prognosis and advanced disease in CRC, such as cribriform growth [14–17]. There is some information on molecular features associated with cribriform glands in CRC, including CpG island methylation and microsatellite instability [15]. However, biological mechanisms explaining the growth pattern and the associated adverse prognosis are unknown.

Diverse genetic and histological differences and similarities between primary tumors and metastases have been reported, and their evaluation may contribute to prognostic stratification. For example, a difference in immune contexture or mutation status between primary tumor and metastasis can predict poor prognosis in CRC [18, 19]. On the other hand, the discordance of mutation statuses of primary tumors and metastases has highlighted the uncharted and nonlinear path of metastatic progression [20–23]. The concepts of heterogeneity of carcinoma tissue and clone selection during the metastatic process may bring some explanation for the differences between primary tumor and metastasis. Since anoikis resistance is a prerequisite for metastasis formation, ability for a high level of anoikis resistance might serve as a stronger selective factor for metastasizing cells than other biological properties present in cancer cell subpopulations. This hypothesis would be supported by enrichment of putative AR structures in metastatic tissue. Indeed, studying matching primary tumors and metastases is needed to better understand essential characteristics of metastatic disease [24].

The prognostic value of putative AR structures in the primary tumors of CRC and the essential role of anoikis resistance in the formation of metastasis prompted us to analyze these structures in LN metastases of CRC. We first aimed to compare their abundance in LN metastases to that in primary tumors. Since we found that the putative AR structures were largely enriched in LN metastases, we analyzed the clinicopathologic features that were associated with such enrichment. Finally, we also sought to assess the prognostic significance of putative AR structures in LN metastases, as well as the possible cumulative prognostic effect of their abundance in primary tumor and in LN metastasis.

## Materials and methods

### Patients

This study was based on a series of 149 CRC patients [25] operated in Oulu University Hospital 2006–2010 (Table 1). Due to deficient sample material, 12 cases were excluded, reducing the number of total cases to 137. In three cases only tumor deposits without nodal metastases were present, and these cases were not included in analyses. LN metastases were found in 57 cases. Metastases composed of purely mucinous growth and totally necrotic metastases were excluded. After these exclusions, we assessed 45 cases with a total of 183 nodal metastases.

**Table 1:** Clinical and pathological features of colorectal carcinoma cases with (N +) and without (N −) LN metastasis

	All	N + (n = 45)	N − (n = 80)
Age, mean (SD)	66.8 (11.2)	65.3 (11.9)	68.5 (9.9)
≤ 65	59 (38.3%)	21 (46.7%)	26 (32.5%)
> 65	90 (61.7%)	24 (53.3%)	54 (67.5%)
Sex			
Male	80 (53.7%)	26 (57.8%)	44 (55.0%)
Female	69 (46.3%)	19 (42.2%)	36 (45.0%)
Primary tumor location			
Proximal	49 (32.9%)	11 (24.4%)	30 (37.5%)
Distal	28 (18.8%)	10 (42.2%)	15 (18.8%)
Rectum	72 (48.3%)	24 (53.3%)	35 (43.8%)
WHO grade			
G1 (Well differentiated)	21 (14.1%)	4 (8.9%)	13 (16.3%)
G2 (Moderately differentiated)	108 (72.5%)	32 (71.1%)	59 (73.8%)
G3 (Poorly differentiated)	19 (12.8%)	9 (20.0%)	8 (10.0%)
TNM stage			
Stage I	27 (18.1%)		24 (30.0%)
Stage II	55 (36.9%)		50 (62.5%)
Stage III	46 (30.9%)	35 (77.8%)	3 (3.8%)^a^
Stage IV	19 (12.8%)	10 (22.2%)	3 (3.8%)
T			
T1	5 (3.4%)	1 (2.2%)	2 (2.5%)
T2	29 (19.6%)	4 (8.9%)	22 (27.5%)
T3	103 (69.6%)	34 (75.6%)	54 (67.5%)
T4	11 (7.4%)	6 (13.3%)	2 (2.5%)
Metastasis (M)			
Yes	19 (12.8%)	10 (22.2%)	3 (3.8%)
No	129 (87.2%)	35 (77.8%)	77 (96.3%)
LN metastasis			
Yes	57 (38.8%)	45 (100%)	
No	90 (61.2%)		80 (100%)
Number of LN metastases, mean (SD)		5.59 (7.65)	
Lymphatic invasion			
Yes	61 (42.1%)	34 (75.6%)	18 (22.5%)
No	84 (57.9%)	11 (24.4%)	62 (77.5%)
Blood vessel invasion			
Yes	27 (18.6%)	16 (35.6%)	7 (8.8%)
No	118 (81.4%)	29 (64.4%)	73 (91.3%)
Infiltrating border			
Yes	43 (29.1%)	19 (42.2%)	14 (17.5%)
No	105 (70.9%)	26 (57.8%)	66 (82.5%)
Cancer type			
Conventional adenocarcinoma	115 (77.2%)	34 (75.6%)	61 (76.3%)
Serrated adenocarcinoma	34 (22.8%)	11 (24.4%)	19 (23.8%)
Mismatch repair (MMR)			
Proficient	137 (92.6%)	44 (97.8%)	72 (90.0%)
Deficient	11 (7.4%)	1 (2.2%)	8 (10.0%)
*BRAF* mutation			
Yes	13 (9.6%)	4 (8.9%)	6 (7.5%)
No	123 (90.4%)	41 (91.1%)	74 (92.5%)
*KRAS* mutation			
Yes	34 (25.0%)	12 (26.7%)	18 (22.5%)
No	102 (75.0%)	29 (64.4%)	56 (70.0%)

^a^Cases with tumor deposits and no LN metastasis

Clinical data and follow-up data were collected from the clinical records and Statistics Finland (Helsinki, Finland). Cancer‐specific survival (CSS) was defined as time from operation to cancer‐related death. The Ethics Committee of Oulu University Hospital had approved this research project (58/2005, 184/2009).

### Histology

General histological assessments of the primary tumors including grading and stage determination were based on whole slide histopathological H&E stained sections [25]. For the assessment of putative AR structures in the primary tumors, we used H&E stained tissue microarrays (TMAs) [25], and these findings have been published [13]. For the primary tumors, only cores from the bulk were assessed. The stained sections of the TMAs as well as metastasis whole slides were digitized for analysis (Leica‐Aperio AT2; Leica Biosystems).

For each LN metastasis and tumor deposit, the most representative section showing the metastasis at its largest dimensions was selected, and all available metastases were assessed. Areal proportions showing complete necrosis and desmoplastic stromal reaction were estimated as percentage of the total metastasis area.

### Detection and quantification of putative AR structures

Assessment of putative AR structures in the primary tumors [13] and the LN metastases and tumor deposits was similarly done by a specialist in anatomical pathology (TTM), blinded for any clinical data. Similarly, the investigator was blinded for pathological observations of the primary tumors when studying LN metastases and vice versa.

**Fig. 1:**
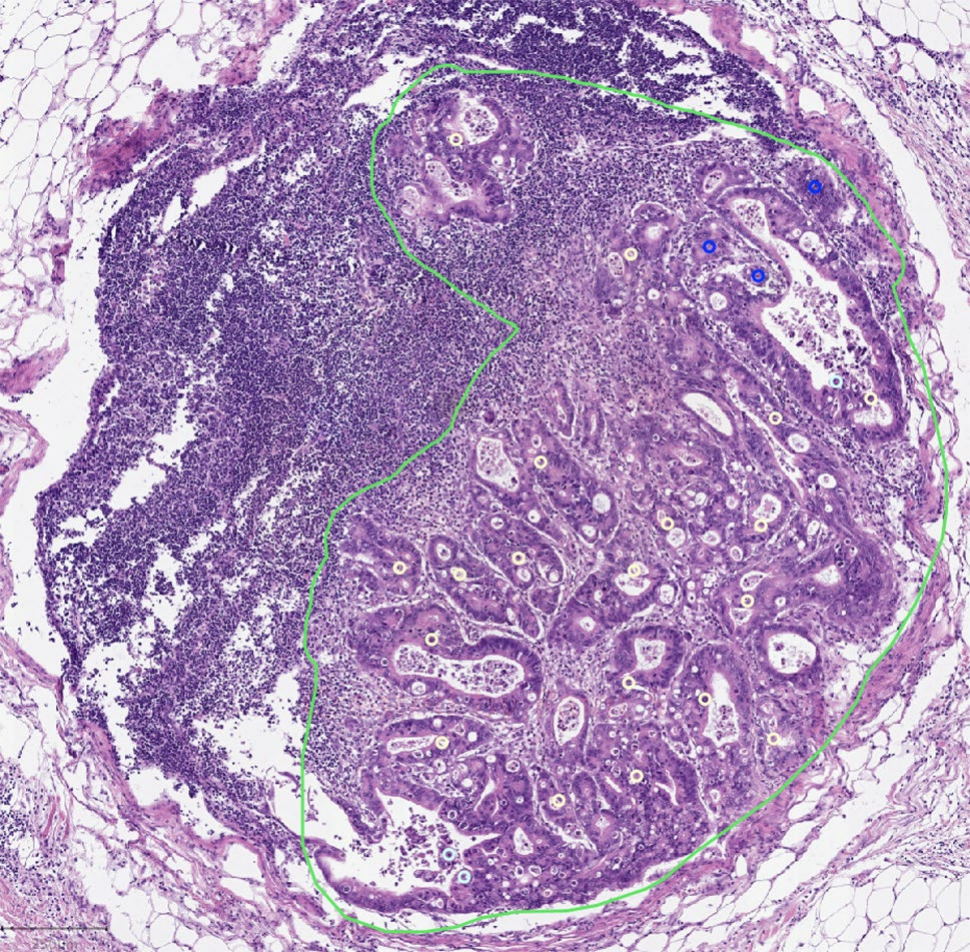
Quantitation of areal density of putative AR structures. An example of LN metastasis of colorectal carcinoma with annotation of metastasis area (green) and putative anoikis-resistant structures (blue solid, cream cribriform and turquoise micropapillary). Scale bar = 250 μm

**Fig. 2:**
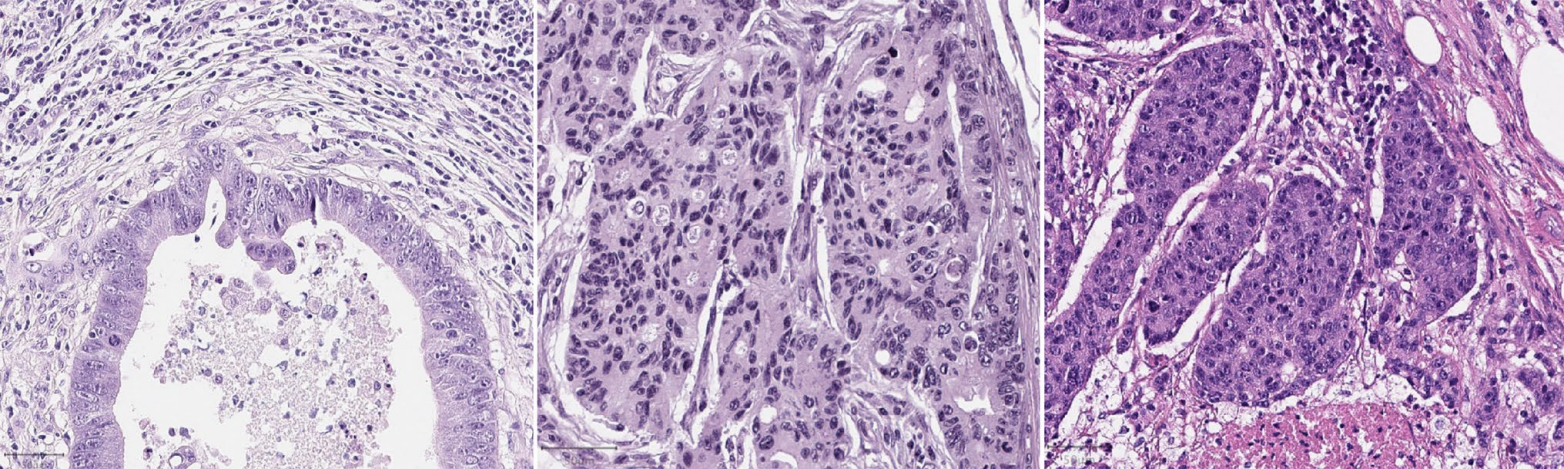
Microphotograph of micropapillary (left), cribriform (center) and solid (right) structures in metastases. Scale bar = 50 μm

We used our previously defined criteria for identification of the three putative AR structures in primary tumor TMAs [13] and metastases using virtual whole slide images (WSIs) of H&E-stained sections (Figs. 1 and 2):

(i) MIPs are cells piled up at the luminal side of the glandular structures, the minimum thickness of this pile being two cells, and the lateral extent at minimum two cells.
(ii) Cribriform structures are groups of cells at least four cells in diameter, and containing scattered, empty spaces without cells.
(iii) Solid structures consist of groups of cells at least four cells in diameter forming solid sheets.

In primary tumors, the area (mm^2^) occupied by the carcinoma was first determined. For determination of area of LN metastases and tumor deposits, complete necrosis, mucin pools and tumor budding at the outer border were excluded. For quantification of putative AR structures, their each occurrence was visually identified in WSIs of H&E-stained sections by using using an image analysis software QuPath (version 0.1.2) [26], and their areal density (structures/mm^2^ of tumor tissue) was computed for each lesion (Fig. 1).

### Statistical analysis

For statistical analysis, IBM SPSS Statistics, version 26 (IBM Corp., Armonk, NY, USA), was used. As the distributions of areal densities of different subpopulations in primary tumors and LN metastasis were skewed, we applied nonparametric Mann–Whitney or Kruskal–Wallis tests to assess their association with clinical and pathological features. Spearman rank correlation (rank correlation coefficient = ρ) for correlation analyses, and Wilcoxon signed rank test for comparing putative AR structure densities between primary tumors and LN metastases. To find optimal cut-off values of areal densities for survival analyses, we utilized receiver operating characteristics (ROC) curve by using the Youden index [27]. For univariate survival analysis, we created Kaplan–Meier curves for cancer-specific survival (CSS) and disease-free survival (DFS). Log-rank test was used to evaluate the statistical significance between survival curves. To express variability of LN AR areal density at case level, standard deviation (SD) was used. Cox regression models were used to analyze the independent prognostic effects of the structure density on CSS when adjusted for covariates. Because of the low number of cases for multivariate analyses, we used models for one covariate at a time, as described in previous literature [13, 28, 29]. A two‐tailed, exact p value ≤ 0.05 was considered statistically significant.

## Results

### Detection of putative anoikis-resistant subpopulations in LN metastases and tumor deposits

All three types of putative AR structures including MIPs, cribriform and solid structures could be identified in LN metastases and tumor deposits of CRC (Figs. 1 and 2), without any visible differences in their histological features as compared to those seen primary tumors [13]. We assessed the areal density (structures/mm^2^ tumor tissue) of each of the 3 putative AR structure types. We also pooled the three components together to derive a sum of areal densities of all three putative AR structure subtypes. For cases with more than one metastasis, the mean of the values was calculated.

### Comparison of areal density of putative anoikis-resistant structures in primary tumor, tumor deposits and LN metastases

To understand better the prognostic effect of high areal density of putative AR structures in primary tumor and to see whether there is any evidence for regulation in a case specific pattern, we assessed the relationship of areal densities in primary tumors and LN metastases. The areal densities of putative AR structures in LN metastases, and in primary tumors separately in cases with (N +) and without nodal metastases (N −) are summarized in Table 2. In primary tumors, no significant differences in putative AR structure counts between N + or N − cases were seen. In comparison of primary tumor and LN metastases, areal densities for cribriform and solid structures as well as for the sum of all three structure types (total putative AR structures, total AR), values were significantly higher in LN metastases than in primary tumors (Table 2, Wilcoxon signed rank test). For MIPs, there was no difference.

**Table 2:** Areal densities of putative AR structures in primary tumors with (N +) and without (N −) LN metastases and in metastatic LN

Putative AR structure	Primary tumor N − median (IQR)	Primary tumor N + median (IQR)	LN metastasis median (IQR)	p value (LN metastasis vs. primary tumour N +)
MIP/mm^2^	1.30 (0.64–2.56)	1.45 (0.82–2.13)	1.67 (0.36–3.33)	0.42
Cribriform/mm^2^	1.55 (0.55–2.55)	1.51 (0.63–2.78)	2.74 (1.81–5.65)	< 0.001
Solid/mm^2^	0.63 (0.27–1.50)	0.63 (0.19–1.88)	1.28 (0.33–2.97)	0.043
Total AR/mm^2^	4.48 (2.74–6.13)	4.76 (3.42–6.65)	6.86 (5.29–10.0)	0.024

Last column presents the Wilcoxon signed rank test p values

Correlations between putative AR structure densities in primary tumors and corresponding LN metastases are summarized in Table 3. Mostly, there were positive correlations between putative AR structure abundances in primary tumors and LN metastases, including MIPs (ρ = 0.37, p = 0.013) and solid structures (ρ = 0.69, p < 0.001). The abundance of cribriform structures in primary tumors correlated with that of solid structures in metastases (ρ = 0.33, p = 0.026) as well. Cribriform and solid structures in primary tumors correlated with total AR in LN metastases (ρ = 0.31, p = 0.036; ρ = 0.36, p = 0.016). However, total AR did not correlate between primary tumors and LN metastases (ρ = 0.24, p = 0.113).

**Table 3:** Spearman correlation of areal density of putative AR structures in primary tumor (N +) and corresponding LN metastasis, and within LN metastases

	Primary MIP	Primary cribriform	Primary solid	Primary total AR
LN metastasis MIP	0.37	− 0.15	− 0.25	− 0.19
	p = 0.013	0.341	0.092	0.216
LN metastasis cribriform	− 0.17	0.24	0.09	0.01
	0.254	0.117	0.569	0.973
LN metastasis solid	− 0.21	0.33	0.69	0.58
	0.170	0.026	< 0.001	< 0.001
LN metastasis total AR	− 0.17	0.31	0.36	0.24
	0.280	0.036	0.016	0.113

	LN metastasis MIP	LN metastasis cribriform	LN metastasis solid
LN metastasis MIP	–		
LN metastasis cribriform	0.07	–	
	p = 0.668		
LN metastasis solid	− 0.15	0.27	–
	0.314	0.069	
LN metastasis total AR	0.27	0.77	0.62
	0.073	< 0.001	< 0.001

In primary tumors, a negative correlation between MIPs and solid structures was observed (ρ = − 0.23, p = 0.006), as well as a positive correlation between solid and cribriform structures (ρ = 0.20, p = 0.017) [12]. Within LN metastases (lower set in Table 3), only a tendency for positive correlation between cribriform and solid structures was detected (ρ = 0.27, p = 0.069).

Tumor deposits were observed in 8 cases with a total number of 20 deposits. Out of 8 cases, 5 cases had both nodal metastases and tumor deposits. For the 20 tumor deposits, median and IQR values for areal density of MIPs were 0 (0–0.35), for cribriform 2.30 (0.18–6.70), solid structures 4.31 (1.39–8.14) and total AR 10.2 (5.85–14.5). Areal density of MIPs was lower than in primary tumors and LN metastases (Table 2, p = 0.028, p = 0.043). Density of cribriform structures in tumor deposits was higher than in primary tumors (p = 0.025), but comparable with that of LN metastases (p = 0.5). Solid structure and total AR densities did not differ from those in LN metastases or primary tumors (p = 0.21–0.5). A correlation in areal density of solid structures was found between tumor deposits and LN metastases (ρ = 0.9, p = 0.037) and also between tumor deposits and primary tumors (ρ = 0.79, p = 0.02). The areal density of MIPs in tumor deposits correlated with MIPs in primary tumors (ρ = 0.755, p = 0.031) and correlated inversely with solid structures in primary tumors (ρ = -0.755, p = 0.031). There was no correlation between total putative AR densities in tumor deposits and primary tumors (ρ = 0.048, p = 0.911) or LN metastases (ρ = 0.2, p = 0.747).

### Variation of putative AR structure areal densities in LN metastases and the effect of extranodal growth

To further assess whether formation of putative AR structures is a random or regulated phenomenon we evaluated variation of the amount of putative AR structures between different metastases of individual patients. To compare intra-case and inter-case variations we calculated standard deviations (SD) for (i) variation within cases (the mean SD of the 45 intra-case SDs), and (ii) overall variation in LN metastases (SD of all 183 nodal metastases). Intra-case SD was lower than overall variation for all types of putative AR structures and for total AR: 2.23 vs 3.56 for MIPs, 2.89 vs 7.72 for cribriform structures, 1.86 vs 4.75 for solids and 5.1 vs 7.76 for total AR. Hence, it seems that AR is comparatively constant within different LN metastases of a single case, when compared to the variability of AR structure density in LN metastases overall.

Since extranodal growth in LN metastases indicates adverse prognosis [5], we were interested to assess possible association of extranodal growth and putative AR structures. Presence of extranodal growth (61/183 LN metastases, 33.3%) associated with a larger metastasis area (Mann–Whitney U = 2743, p = 0.02) and with the higher areal density of solid structures (U = 3011, p = 0.024), but not significantly with that of MIPs, cribriform or total putative AR densities were seen (U = 3143, p = 0.061; U = 3720, p = 0.857; U = 3472, p = 0.3).

### Putative AR structures in LN metastases and clinicopathological features

The relationship of areal densities of the three putative AR structure types and total putative AR areal density in nodal metastases and clinicopathological features is summarized in Table 4. Proximal location of primary tumor associated with lower number of MIPs in LN metastasis, with distal location associating with the highest density (p = 0.005). Solid structures showed a tendency to be more abundant in proximal disease as compared to distal (p = 0.061). WHO grade 1 in primary tumors associated with lower total AR density, than grade 2 tumors (p = 0.027). *BRAF* V600E-mutation in primary tumor associated with abundance of solid structures (p = 0.011) and with a tendency for less MIPs (p = 0.099). Also, a tendency for more MIPs with the presence of blood vessel invasion in primary tumors was observed (p = 0.058).

**Table 4:** Relationship between clinicopathological features and the areal densities of structures representing different putative anoikis-resistant populations (MIPs, cribriform, and solid) and the sum of areal densities of all putative anoikis-resistant subpopulations (total areal density) in metastatic LN

	MIP	Cribriform	Solid	Total AR
	Median (IQR)	Median (IQR)	Median (IQR)	Median (IQR)
Age 65				
p-value	0.838	0.699	0.569	0.495
< 65	1.42 (0.36–3.37)	2.52 (1.85–4.51)	1.22 (0.35–2.88)	6.44 (5.55–8.54)
> 65	1.76 (0.94–3.23)	3.30 (2.09–6.17)	1.42 (0.41–2.97)	8.13 (4.47–10.29)
Sex				
p-value	0.827	0.696	0.629	0.872
Male	1.54 (0.36–3.65)	2.89 (1.75–5.51)	1.44 (0.45–3.53)	6.51 (5.20–10.28)
Female	1.74 (0.36–3.22)	2.68 (1.85–6.92)	0.94 (0.24–2.95)	7.30 (5.55–8.89)
Tumor location				
p-value	0.005	0.124	0.061	0.183
Proximal	0.36 (0.14–1.09)	2.74 (1.42–5.78)	3.58 (0.55–5.61)	6.86 (4.55–9.84)
Distal	3.25 (1.74–4.08)	5.54 (2.57–7.82)	0.77 (0.24–1.52)	9.21 (7.04–11.42)
Rectum	1.73 (0.55–3.51)	2.46 (1.77–4.39)	1.25 (0.33–2.32)	6.48 (4.81–9.38)
T				
p-value	0.789	0.830	0.738	0.932
T1	3.37^a^	1.93^a^	1.28^a^	6.59^a^
T2	2.17 (0.93–3.86)^b^	2.54 (2.08–4.80)^b^	0.49 (0.19–1.88)^b^	8.27 (5.01–8.72)^b^
T3	1.41 (0.33–3.46)	3.30 (1.75–5.51)	1.30 (0.45–3.54)	6.95 (5.23–10.28)
T4	1.52 (1.17–1.94)	3.51 (2.32–6.85)	1.44 (0.24–1.57)	6.24 (5.34–9.54)
TNM stage				
p-value	0.702	0.120	0.397	0.397
I				
II				
III	1.74 (0.33–3.46)	2.57 (1.76–5.35)	1.01 (0.31–3.00)	6.71 (5.20–10.22)
IV	1.29 (0.36–1.96)	4.37 (2.52–6.85)	1.55 (1.32–2.88)	8.20 (6.44–9.84)
WHO grade				
p-value	0.112	0.424	0.208	0.027
G1	1.52 (0.69–1.81)^b^	2.39 (2.04–2.48)^b^	0.25 (0.15–0.83)^b^	3.95 (3.06–4.95)^b^
G2	2.16 (0.79–3.55)	3.64 (1.85–6.31)	1.30 (0.40–3.27)	8.13 (6.18–10.32)
G3	0.36 (0.33–1.17)	2.68 (1.85–4.51)	1.52 (0.93–2.88)	6.44 (5.55–8.54)
Lymphatic invasion				
p-value	0.927	0.327	0.176	0.137
Yes	1.54 (0.36–3.29)	3.39 (1.85–6.83)	1.39 (0.35–3.54)	7.56 (6.05–10.28)
No	2.56 (0.33–3.37)	2.57 (1.76–3.72)	0.55 (0.17–2.04)	6.59 (3.97–8.00)
Blood vessel invasion				
p-value	0.058	0.507	0.255	0.652
Yes	2.80 (1.27–3.87)	3.04 (1.57–4.44)	1.25 (0.22–1.55)	6.50 (5.62–9.65)
No	1.09 (0.33–2.60)	2.74 (2.32–5.78)	1.35 (0.55–3.00)	8.00 (5.34–10.22)
Tumor border				
p-value	0.748	0.334	0.093	0.175
Pushing	1.68 (0.45–3.29)	2.62 (1.77–3.84)	0.93 (0.31–1.66)	6.65 (4.39–8.54)
Infiltrating	1.67 (0.36–3.65)	4.43 (2.32–6.85)	2.04 (0.45–4.03)	8.89 (6.21–10.37)
Cancer type				
p-value	0.176	0.825	0.706	0.558
Conventional	1.73 (0.48–3.46)	2.86 (1.93–5.78)	1.30 (0.45–2.95)	6.95 (6.05–9.84)
Serrated	1.09 (0.11–2.39)	2.74 (1.76–5.51)	0.93 (0.15–3.53)	6.44 (4.38–10.28)
*KRAS*				
p-value	0.966	0.274	0.641	0.524
*KRAS* mut	1.56 (1.02–3.00)	4.54 (2.26–6.84)	0.84 (0.38–1.81)	8.72 (4.97–10.11)
wt	1.78 (0.36–3.46)	2.49 (1.76–4.24)	1.28 (0.31–2.95)	6.57 (5.23–8.79)
*BRAF*				
p-value	0.099	0.577	0.011	0.203
*BRAFwt*	1.74 (0.48–3.37)	2.68 (1.85–5.51)	1.22 (0.31–2.59)	6.71 (5.23–9.77)
*BRAF V600E*	0.34 (0.16–1.38)^b^	4.44 (2.21–6.98)^b^	4.70 (2.55–7.04)^b^	9.27 (7.35–12.97)^b^
Mismatch repair				
p-value	0.444	0.178	0.578	0.311
Deficient	0.33^a^	8.38^a^	2.88^a^	11.58^a^
Proficient	1.70 (0.40–3.33)	2.71 (1.81–5.43)	1.28 (0.33–2.97)	6.78 (5.29–9.81)

Mann–Whitney and Kruskal–Wallis tests were used

^a^Category had one case, quartiles could not be presented, so actual numbers are presented

^b^Category had 4 cases

### Putative AR structures in LN metastases and survival

**Fig. 3:**
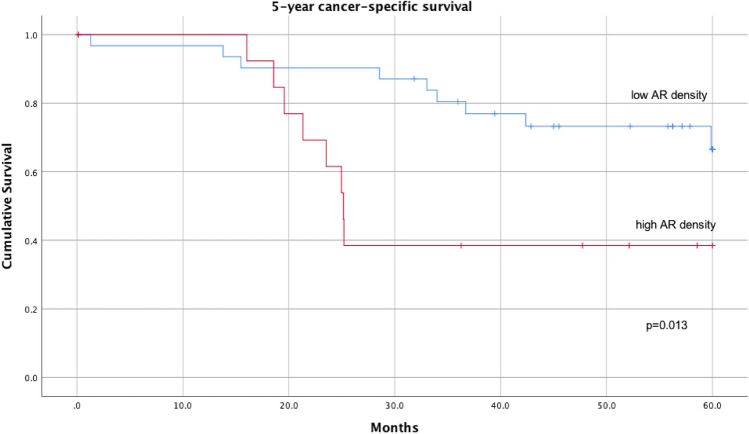
Kaplan–Meier curve showing cancer‐specific survival (60 months; log rank) in patients (n = 45) with high (n = 14) and low (n = 31) areal densities of the putative anoikis‐resistant structures in LN metastases (cut-off 9,2 structures/mm^2^)

As we have previously shown, that high areal density of putative AR structures is an independent indicator of adverse prognosis [13], we expected similar association for AR structures in metastases. For metastases, optimal cut-off value for low and high areal density of putative AR structures in terms of survival was determined with Youden index analysis of ROC curve plotted on survival. Optimal cut-off for the total AR areal density was 9.2; for MIPs 0.23; cribriforms 5.4, and for solids 1.25 structures/mm^2^. In univariate analysis for 5-year cancer specific survival, high total AR density (p = 0.013; Fig. 3) and cribriform density (p = 0.011) associated with worse prognosis, while for MIP (p = 0.119) and solid structure densities (p = 0.051) there was no statistically significant association. For 5-year disease-free survival only cribriform density showed a significant association (p = 0.003).

Due to the setting where only nodal metastatic cases were analyzed limiting the number of cases, multivariate evaluation of the independent prognostic value included only the major prognostic factors. Accordingly, the Cox regression model was adjusted for tumor stage (T1‐2 vs T3‐4), N stage (1 vs. 2), and presence or absence of distant metastasis (Table 5). High total putative AR density in nodal metastases showed independent prognostic value for cancer specific survival except when compared to distant metastasis (M), as elevated hazard ratio was non-significant only when adjusted for distant metastasis [p = 0.075, HR 2.53 (0.91–7.03)]. However, we did not observe evidence for an association between distant metastasis and total putative AR density (data not shown), suggesting that distant metastasis is not a true confounder. Also, total putative AR density in metastases was a stronger prognostic factor than that of the primary tumor (Table 5, Model 1: HR 2.95 (1.08–8.07) p = 0.035 vs. HR 1.06 (0.997–1.12), p = 0.063).

**Table 5:** Cox regression models for the independent prognostic significance of high Total AR structure count (> 9.2/mm^2^) in LN metastases (CSS; 5 year)

Covariates	Univariate	Model 1	Model 2	Model 3	Model 4	Model 5
		Primary tumor total AR count	T Stage	M	N stage	Location
	HR (95% CI)	HR (95% CI)	HR (95% CI)	HR (95% CI)	HR (95% CI)	HR (95% CI)
LN Metastasis	3.25 (1.23–8.60)	2.95 (1.08–8.07)	4.00 (1.36–11.8)	2.53 (0.91–7.03)	3.53 (1.30–9.56)	3.25 (1.22–8.60)
Total AR count	**p = 0.018**	**p = 0.035**	**p = 0.012**	p = 0.075	**p = 0.013**	**p = 0.018**
> 9.2/mm^2^						
Primary tumor	1.07 (1.01–1.14)	1.06 (0.997–1.12)				
Total AR count	**p = 0.023**	p = 0.063				
> 6.86/mm^2^						
T 1–2 vs 3–4	0.75 (0.21–2.60)		0.43 (0.11–1.72)			
	p = 0.643		p = 0.234			
M	4.42 (1.62–12.0)			3.57 (1.26–10.1)		
	**p = 0.004**			**p = 0.017**		
N 1 vs 2	1.39 (0.53–3.60)				1.64 (0.62–4.32)	
	p = 0.502				p = 0.318	
Location rectum/colon	1.08 (0.42–2.79)					0.96 (0.37–2.49)
	p = 0.881					p = 0.925

In Models 1–5, LN Metastasis Total AR count was adjusted with one covariate at a time due to insufficient number of cases for multivariate analysis

Bold values indicate statistically significant P values

Univariate column presents the crude value

*HR* Hazard ratio

### Loss or gain of areal densities of putative AR structures in LN metastases and prognosis

**Fig. 4:**
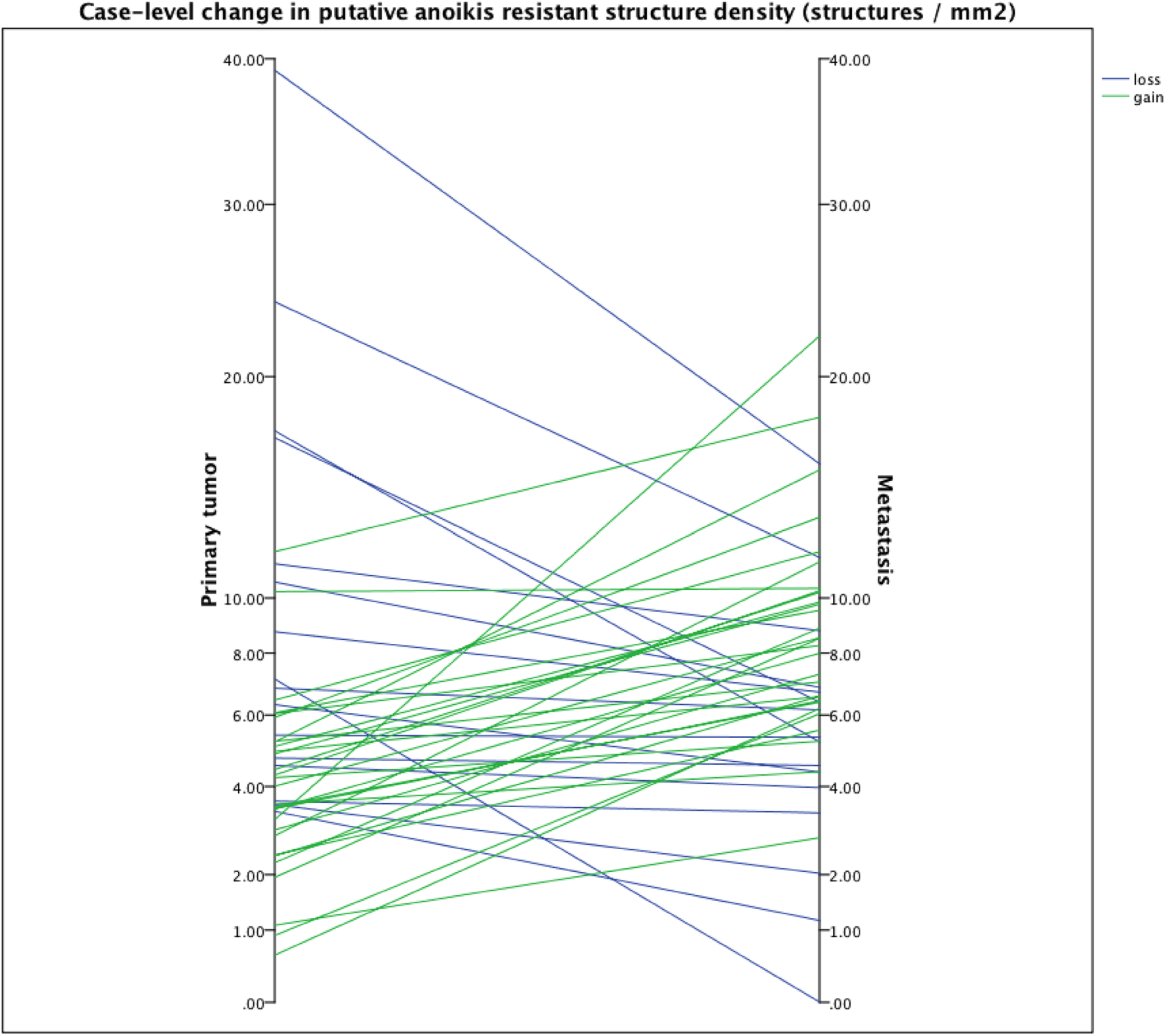
Parallelogram showing gain (n = 29) or loss (n = 16) of areal densities of putative anoikis-resistant structures in 45 primary tumors and the corresponding LN metastases

Since high areal density of putative AR structures in LN metastasis was associated with adverse prognosis (see above), we hypothesized that increase of putative AR density in metastasis as compared with primary tumor would indicate effective selection of highly AR clones capable of further dissemination of cancer and thereby poor prognosis. Accordingly, we compared prognosis in groups with either gain or loss of putative AR areal density in metastases as compared with that in the primary tumor. Total putative AR density was higher in LN metastases than in primary tumors in 29 cases out of 45 (64.4%) (Fig. 4). Among cases with a gain in total AR, mean total AR value in primary tumors was 4.26 and 9.13 in LN metastasis. Among the cases with a loss of total AR, values were 10.85 in primary tumors and 5.76 in LN metastasis. Clinicopathological features in patients with gain or loss of total AR density in metastasis are shown in Table 6. Gain was more prevalent in cancers of distal colon (Table 6; p = 0.008) and loss more prevalent in proximal colon, but no other differences emerged. Gain or loss did not have any effect on survival (data not shown).

**Table 6:** Comparison of clinicopathological features in CRC with loss or gain of putative AR density in LN metastases

Feature	AR gain (n = 29)	AR loss (n = 16)	p value
Total AR/mm^2^			
Primary tumor	4.26	10.85	
LN Metastasis	9.13	5.76	
Age			
≤ 65	13 (44.8%)	8 (50%)	0.311
> 65	16 (55.2%)	8 (50%)	
Mean (SD)	66.28 (13.0)	63.5 (9.80)	0.742
Sex			
Male	15 (51.7%)	11 (68.8%)	0.274
Female	14 (48.3%)	5 (31.3%)	
Tumor location			
Proximal	3 (10.3%)	8 (50%)	**0.008**
Distal	9 (31.0%)	1 (6.3%)	
Rectum	17 (58.6%)	7 (43.8%)	
WHO grade			
G1	1 (3.4%)	3 (18.8%)	0.156
G2	23 (79.3%)	9 (56.3%)	
G3	5 (17.2%)	4 (25%)	
TNM stage			
Stage I			0.681
Stage II			
Stage III	22 (75.9%)	13 (81.3%)	
Stage IV	7 (24.1%)	3 (18.8%)	
T			
T1	1 (3.4%)		0.682
T2	3 (10.3%)	1 (6.3%)	
T3	21 (72.4%)	13 (81.3%)	
T4	4 (13.8%)	2 (12.5%)	
Metastasis (M)			
Yes	7 (24.1%)	3 (18.8%)	0.737
No	22 (75.9%)	13 (81.3%)	
Local nodal metastases			
Mean (SD)	5.37 (5.03)	6.00 (11.37)	0.128
Lymphatic invasion			
Yes	23 (79.3%)	11 (68.8%)	0.532
No	6 (20.7%)	5 (31.3%)	
Blood vessel invasion			
Yes	10 (34.5%)	6 (37.5%)	0.841
No	19 (65.5%)	10 (62.5%)	
Infiltrating border			
Yes	11 (37.9%)	8 (50%)	0.438
No	18 (62.1%)	8 (50%)	
Cancer type			
Conventional adenocarcinoma	23 (79.3%)	11 (68.8%)	0.523
Serrated adenocarcinoma	6 (20.7%)	5 (31.3%)	
Mismatch repair (MMR)			
Proficient	29 (100%)	15 (93.8%)	0.356
Deficient		1 (6.3%)	
*BRAF* mutation			
Yes	3 (10.3%)	1 (6.3%)	0.715
No	26 (89.7%)	15 (93.8%)	
*KRAS* mutation			
Yes	9 (34.6%)	3 (20%)	0.419
No	17 (65.4%)	12 (80%)	

p values for chi-square or Fisher’s exact test are presented

### Prognostic effect of cumulative amount of putative AR structures in primary tumors and in LN metastases

**Fig. 5:**
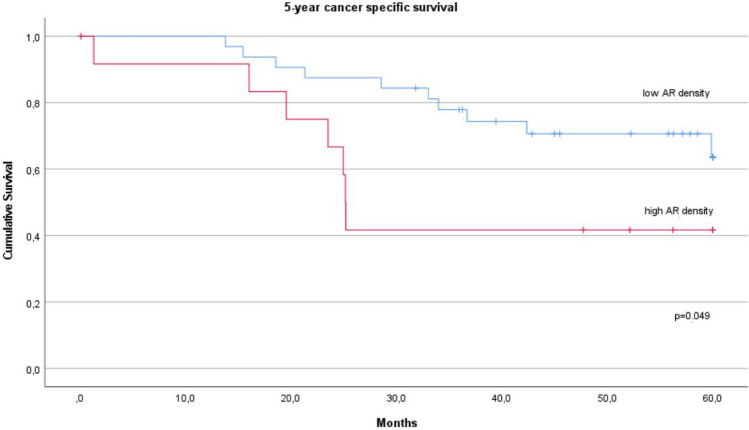
Kaplan–Meier curve showing cancer‐specific survival (60 months; log rank) in patients (n = 45) with high (n = 13) and low (n = 32) cumulative areal densities of the putative anoikis-resistant structures in tumor tissue in primary tumor and in LN metastases (cut-off 15,5 structures/mm^2^)

Since abundance of putative AR structures in primary tumor and LN metastasis both were similarly associated with adverse prognosis, we were interested to look for possible cumulative survival effect of putative AR abundance in these anatomical locations. After calculating the sum of the areal densities of the three putative AR structure types in primary tumors and in metastases, we determined the optimal cut-off by using the ROC curve by applying Youden index method (15,5 structures/mm^2^). A significant association with poorer CSS at 5 years was found for the high cumulative (primary tumor and LN metastasis) total AR density (p-value of log rank test 0.049; Fig. 5).

Multivariate evaluation of the independent prognostic value of high cumulative total putative AR density by Cox regression model is shown in Table 7. In contrast to high total putative AR in LN metastases, a high cumulative total putative AR density in primary tumor and nodal metastases showed an independent prognostic value for CSS also when compared to distant metastasis (M) [p = 0.035, 2.90 (1.08–7.82)] in addition to T stage and N stage.

**Table 7:** Cox regression models for the independent prognostic significance of cumulative high Total AR structure count (> 15.5/mm^2^) in primary tumors and LN metastases (CSS; 5 year)

Covariates	Univariate	Model 1	Model 2	Model 3	Model 4
		T Stage	M	N stage	Location
	HR (95% CI)	HR (95% CI)	HR (95% CI)	HR (95% CI)	HR (95% CI)
Primary + LN Metastasis	2.57 (0.97–6.80)	3.02 (1.05–8.66)	2.90 (1.08–7.82)	2.73 (1.02–7.33)	2.83 (0.99–8.13)
Total AR count					
> 15,5/mm^2^	p = 0.057	**p = 0.040**	**p = 0.035**	**p = 0.046**	p = 0.052
T 1–2 vs 3–4	0.75 (0.21–2.60)	0.498 (0.13–1.93)			
	p = 0.643	p = 0.313			
M	4.42 (1.62–12.0)		4.89 (1.76–13.6)		
	**p = 0.004**		**p = 0.002**		
N 1 vs 2	1.39 (0.53–3.60)			1.56 (0.59–4.08)	
	p = 0.502			p = 0.369	
Location rectum/colon	1.08 (0.42–2.79)				1.30 (0.46–3.64)
	p = 0.881				p = 0.621

In Models 1–4, LN metastasis Total AR count was adjusted with one covariate at a time due to insufficient number of cases for multivariate analysis

Bold values indicate statistically significant p values

Univariate column presents the crude value

*HR* Hazard ratio

To analyze the contributions of primary tumor and metastasis on the prognostic effects of total AR densities, we divided the cases in into four groups according to putative AR areal density class. Class was determined with the cut-offs used for survival analyses (see above; 6.86/mm^2^ primary tumor; 9.2/mm^2^ LN metastasis):

- Group 1: primary tumor Total AR high, LN metastasis Total AR high (n=4)
- Group 2: primary tumor Total AR low, LN metastasis Total AR high (n=10)
- Group 3: primary tumor Total AR high, LN metastasis Total AR low (n=6)
- Group 4: primary tumor Total AR low, LN metastasis Total AR low. (n=25)

**Fig. 6:**
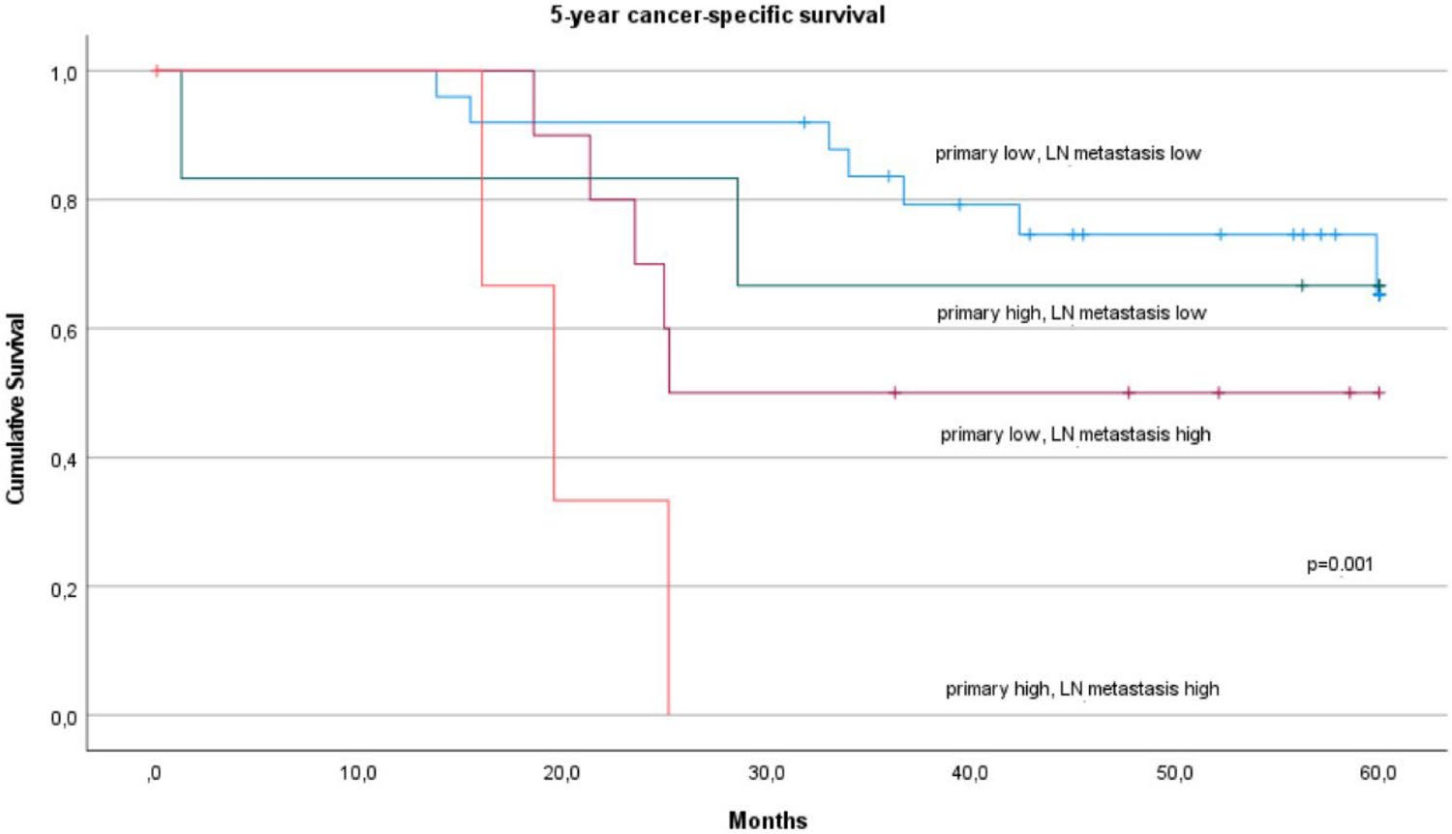
Kaplan–Meier curve showing cancer‐specific survival (60 months; log rank) in patients (n = 45) divided into four groups based on high or low total AR values of primary tumor and LN metastases. Group 1: primary tumor total AR high, LN metastasis total AR high (n = 4), Group 2: primary tumor total AR low, LN metastasis total AR high (n = 10), Group 3: primary tumor total AR high, LN metastasis total AR low (n = 6), Group 4: primary tumor total AR low, LN metastasis total AR low (n = 25). Cut-offs for total AR were 9.2/mm^2^ for LN metastasis, 6.86/mm^2^ for primary tumor

Kaplan–Meier analysis for CSS at 5 years indicates that group 1 has the worst prognosis (p value of log rank test 0.001) when all groups are compared simultaneously (Fig. 6). Hazard ratios for each group against the other three groups were: Group 1, 8.64 (2.18–34.3), p = 0.002; Group 2: 1.75 (0.61–5.02), p = 0.296; Group 3: 0.80 (0.18–3.53), p = 0.773; Group 4: 0.40 (0.15–1.05), p = 0.062. Similarly, for 5-year DFS, group 1 was associated with the worst prognosis (p-value of log rank test was 0.002). Hazard ratios for each group against the other three groups were: Group 1: 16.9 (2.34–122), p = 0.005; Group 2: 0.75 (0.22–2.57), p = 0.645; Group 3: 0.60 (0.14–2.60), p = 0.493; Group 4: 1.03, (0.41–2.63), p = 0.944. These findings for CCS and DFS indicate that high total AR in primary tumors and LN metastases might have a cumulative negative effect on survival.

## Discussion

We have recently shown that cells in micropapillary, cribriform and solid structures in CRC show features indicating resistance to anoikis as these structures are mainly composed of cells without contact with the ECM, but still do not show evidence for increased apoptosis rate [13]. Interestingly, abundance of these putative AR structures in primary tumors is associated with adverse prognosis [13]. To further characterize significance and biology of the putative AR structures, we have here quantified these structures in LN metastases and tumor deposits of CRC. The overall areal density of the putative AR structures was higher in LN metastases suggesting some selection during the formation of metastasis. Since pathogenesis of tumor deposits may differ from that of LN metastases [30], we were also interested to compare these two lesion types harboring disseminated carcinoma cells, but likely related with low number of cases with deposits, no conclusive differences were observed. High areal density of putative AR structures in LN metastases associated with adverse prognosis, and abundance of these structures in both primary tumors and in LN metastases showed a cumulative adverse effect on survival.

Current study is the first one to analyze putative AR structures in LN metastases of carcinoma and to compare occurrence of these structures in the primary tumors. Areal density of the putative AR structures was higher in LN metastases in the majority of cases. Such enrichment of putative AR structures in LN metastasis is consistent with the concept that during formation of metastasis, cell populations that are more capable of forming putative AR structures are positively selected during the formation of metastasis. This indirect evidence suggests that putative AR structures are markers of actual anoikis resistance that provides fundamental advantages during the metastatic cascade, including survival without ECM contact and in improper ECM [6, 31]. We also identified a minor group of cases which showed decrease of areal density of putative AR structures in LN metastases. We found no differences in clinicopathological features or prognosis between cases with loss or gain of putative AR structures in LN metastases, and mechanisms for the loss remain unknown. We speculate that selection of metastasizing cells is not always directly associated with the mechanisms related to anoikis resistance and that it is possible that in some cases, clones that actually metastasize are composed of cells with lower anoikis resistance.

In our analyses, areal densities of putative AR structures were positively correlated between LN metastases and corresponding primary tumors. In addition, variation of areal densities of AR structures was less in different nodal metastases of each patient as compared with variation among metastases of all patients. This suggests that the patterns of putative AR structures are rather stable in each patient. In addition, we found evidence that in each patient different LN metastases show mostly similar patterns of putative AR structures, indicating that the patterns of putative AR structures are rather stable in each patient. Such stability also suggests that the formation of putative AR structures is not solely dependent on the original microenvironment at the primary site. In general, genetic features of metastases can be expected to be relatively stable within a cancer case, since most metastases have been shown to be monophyletic (monoclonal) polyphyly remaining occasional [32]. Our previous in vitro studies have indicated that *BRAF* mutation is one possible inducing factor of AR structures [12]. Supporting this concept, in the current study *BRAF* V600E-mutation in primary tumors associated with abundance of solid structure type of putative AR structures in LN metastases.

High areal density of putative AR structures in LN metastases was a marker of adverse cancer specific survival in CRC. The prognostic effect was independent when adjusted for tumor and node stage. Although the effect did not reach statistical significance when adjusted for distant metastasis [p = 0.075, HR 2.53 (0.91–7.03)], absence of association between the total AR counts and distant metastasis, a dominant prognostic factor, suggests that the effect of putative AR structures might be independent even in terms of distant metastasis. The prognostic effect of putative AR structure amount in LN metastases is a novel finding which should be confirmed with an independent case series. In addition, the current cut-off values for high and low areal density should be considered provisional, likely needing adjustment based on additional case series. However, areal density of putative AR structures could complement the list of metastasis-based prognostic features such as N category, extranodal extension and LN ratio [5, 33, 34]. Interestingly, areal densities of putative AR structures in primary tumor and LN metastasis showed a cumulative prognostic value. Since true AR is a conceptual prerequisite for metastasis [6, 31] such associations with prognosis are biologically plausible. Occurrence of cumulative prognostic effect indicates that the prognostic effect of putative AR density in LN metastases is not solely dependent on features transferred from the primary tumor but might suggest that there is enrichment of some additional aberrations during the progression of the disease.

For advanced stage CRC, only a limited number of predictive biomarkers are available, and validated predictive markers for adjuvant chemotherapy in stage III are lacking [4, 35]. If the prognostic value of areal density of putative AR structures in LN metastases can be confirmed in separate case series, this feature might serve as a clinically useful prognostic factor in stage III CRC. Further studies are also required to evaluate whether it could be used as a predictive factor for specific therapies.

Although our concept of putative AR structures represents a biologically plausible explanation for the prognostic associations we observed, actual anoikis resistance can only be detected in in vitro experiments [12]. Therefore, alternative biological mechanisms for the prognostic effect are possible. It should be noted that many classical morphological features with prognostic value, such as tumor grade, still lack biological explanation. It is of interest that previous structural analyses in CRC have indicated that growth patterns quite similar to putative AR structures, including micropapillary, solid and cribriform growth, and some of the so called poorly differentiated clusters [36, 37] associate with poor prognosis [38]. However, quantification of such components has often been poorly specified and prognostic value is not always straightforward [14]. Taken together, although putative AR structures as a biological concept needs additional verification, their prognostic value is in line with previous studies using different phraseology or biological framework for structural analyses.

Extranodal invasion in LN metastases is a rather novel prognostic factor in several carcinoma types including CRC [5]. We observed only an association with areal density of solid structures, but not with other types or the total putative AR density. It seems likely that extranodal growth and putative AR structures are driven by mainly different set of aberrations, and that the mechanisms of their prognostic effects are mainly different.

Our study represents a comprehensive assessment of the 137 patients of which 57 had LN metastases and 45 were examined. Due to the limited number of metastatic cases, our observations should be confirmed with an independent case series. However our conclusions are supported by systematic analyses of all LN metastases. The assessment was blinded for both clinicopathological features and the results of putative AR structure analyses of the primary tumors. Quantification of areal density using WSIs with proper software tools enables a higher detail of measurement than conventional light microscope analysis. Also, the reproducibility of the quantification method was confirmed in our previous paper [13].

## Conclusions

Our comprehensive analysis of LN metastases in CRC shows that putative AR structures are enriched in LN metastases as compared with primary tumors. As this could be related with a survival benefit of malignant cells linked with true anoikis resistance, the finding lends some support to the concept of putative AR structures being related with true anoikis resistance. Abundance of putative AR structures in nodal metastases is a new marker of poor prognosis.
